# A case of fatal cutaneous caput medusae hemorrhage

**DOI:** 10.1002/ccr3.1996

**Published:** 2019-01-28

**Authors:** Nikolaos Melas, Amil Haji Younes, Peter Magnusson

**Affiliations:** ^1^ Centre for Research and Development Uppsala University/Region Gävleborg Gävle Sweden; ^2^ Cardiology Research Unit, Department of Medicine Karolinska Institutet Stockholm Sweden

**Keywords:** alcoholic liver cirrhosis, caput medusae, esophageal varices, hemorrhage, hypertensive gastropathy

## Abstract

Alcoholic liver cirrhosis leads to portal venous hypertension, which can result in a caput medusae formation. Life‐threatening hemorrhage from a ruptured caput medusae vein is a rare complication. It is crucial to stop the bleeding promptly. A transjugular intrahepatic portosystemic shunt is considered potentially lifesaving.

## 1 BACKGROUND

Patients with liver cirrhosis form varices at the rate of 5%‐15% per year, and one‐third of these subjects experience variceal bleeding.^1^, ^2^ While varices are most commonly localized in the gastroesophageal region, ectopic sites may form at any point where the portal and systemic venous circulations communicate or where anastomoses have been surgically created.^3^ We report the unusual case of a fatal cutaneous hemorrhage from caput medusae.

## 2 CASE PRESENTATION

**Figure 1:**
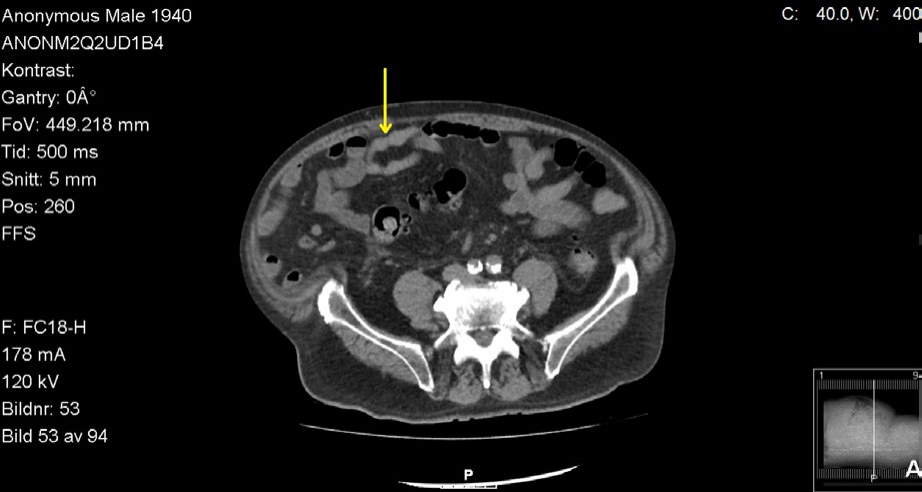
CT without contrast (axial) shows protruding paraumbilical veins

**Figure 2:**
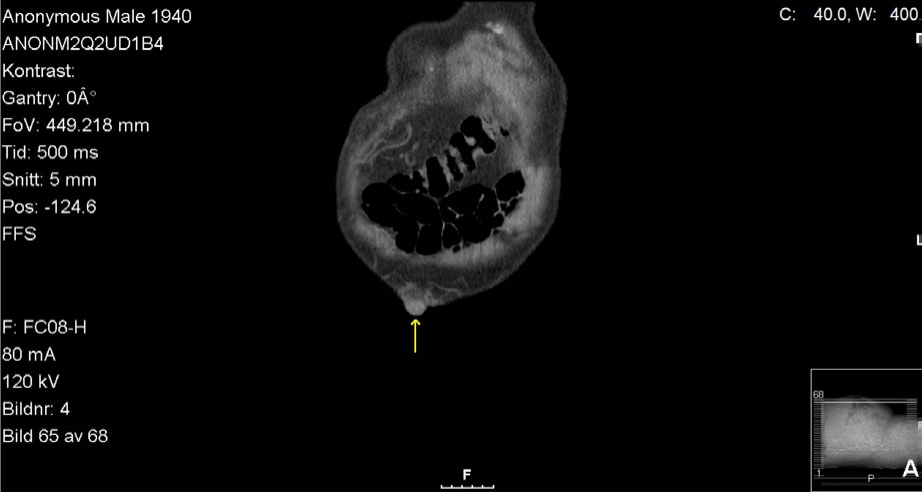
CT without contrast (coronal) shows dilated paraumbilical veins

A 76‐year‐old Caucasian male was followed in the gastroenterology unit because of alcoholic liver cirrhosis (ALC) due to a daily consumption of 0.75 L of wine over the past two decades. He had a history of type 2 diabetes mellitus, hypertension, hyperlipidemia, osteoarthritis, previous cholecystectomy, and carotid endarterectomy of the right common and internal carotid artery. He was diagnosed with ALC two years ago when he presented with an episode of hematemesis and melena. During hospitalization, he underwent esophagogastroduodenoscopy (EGD) that revealed signs of ALC‐decompensation with grade 2 esophageal varices and portal hypertensive gastropathy. Abdominal ultrasound examination confirmed signs of liver cirrhosis. A computerized tomography of the abdomen showed dilatation of the paraumbilical veins (Figures 1 and 2). Blood analysis showed a spontaneously elevated international normalized ratio (INR), hypoalbuminemia, elevated liver enzymes, and anemia. His treatment consisted of a daily dose of omeprazole 20 mg, aldactone 100 mg, propranolol 40 mg, furosemide 40 mg, sodium picosulfate 5 mg, insulin lispro 4 units as needed, and oxazepam 5 mg as needed. After discharge from the hospital, he was followed regularly in our gastroenterology unit. Unfortunately, he continued drinking heavily and over the last year of care, he started taking oxazepam regularly, but without a prescription and at unknown doses. Two years after the ALC diagnosis, he was admitted to the hospital due to a new episode of decompensation with gastrointestinal bleeding and liver encephalopathy. He had signs of portal hypertension, manifesting as distended and engorged superficial epigastric veins radiating from the umbilicus across the abdomen. Three days before admission to the hospital, he abruptly quit drinking which resulted in withdrawal symptoms such as tremor, tachycardia, and anxiety. Now, he had developed liver encephalopathy and was discharged with the instruction that he take lactulose 20 g twice daily and ferrous sulfate 100 mg twice daily. He was also strongly advised to abstain from alcohol. Only 10 days after discharge from the hospital, he was re‐admitted for severe bleeding from a superficial epigastric vein. His wife had found him lying on the floor with impaired consciousness and copious amounts of blood spurting from a distended vessel in the umbilical area. According to the ambulance report, the patient had no abdominal pain prior the episode and suddenly started bleeding from the umbilicus. In the emergency room, a rupture in the caput medusae vessel was revealed, but bleeding could be stopped with pressure bandage. Hemodynamics and respiration were stable after administration of Glypressin (terlipressin) and intravenous fluids. He stated he had not adhered to his prescribed medications and that he had only been taking ferrous sulfate. The next day while still in the hospital, he took a shower, causing the pressure bandage to come loose, resulting in a torrent of blood rushing out of one of the umbilicus vessels. The area was sutured closed under local anesthesia and the bleeding stopped. The patient refused to stay in the hospital and was discharged the next day.

One month later, the patient's wife summoned help because she thought he had died. According to the medical records from the general practitioner who visited patient´s home and confirmed his death, the patient was about as usual the evening before. The wife found him dead the next morning in bed. After physical examination, the physician concluded the cause of death was massive bleeding from a ruptured caput medusae vessel. The volume of blood that was found all over the body, his clothes, in the bed, and on the floor was large, possibly a few liters. When the physician removed the bandage from the vessel and compressed the abdomen on the caput medusae, dark blood poured out from an opening in one of the vessels of the caput medusae. Based on the patient's medical history and prior similar episodes of bleeding at the umbilicus area, the physician determined the cause of death to be fatal bleeding from the caput medusae. An autopsy was deemed unnecessary.

## 3 DISCUSSION

Alcoholic liver disease is the most common liver disease in the Western world.^4^ The spectrum of liver disease varies from simple steatosis to cirrhosis. Risk factors for liver disease progression include the amount and type(s) of alcohol consumed, drinking patterns (outside meals), female sex, malnutrition, and genetic factors.^5^ Portal hypertension is a common complication of liver cirrhosis and develops as a consequence of resistance to portal blood flow.^6^ The primary etiology of portal hypertension is ALC.^7^ Collateral vessels may arise at any point of communication between the portal and systemic venous system and form varices in order to decompress the portal system in the presence of portal hypertension.^8^ Locations for varices may include the coronary, short gastric, esophageal, azygos, and hemorrhoid veins. Chronic portal hypertension increases the distension of these vessels, which also increases their propensity to rupture. In Cruveilhier‐Baumgarten syndrome, the umbilical vein ruptures which, in turn, distends the superficial epigastric veins. Caput medusa is a sign of portal hypertension in that it shows these varices are forming.

The term caput medusae comes from the serpentine appearance of the dilated epigastric veins, which resembles the head (Latin, caput) of the gorgon Medusa in Greek mythology, described as a human female whom the goddess Athena transformed into a monster with venomous snakes in place of hair.^9^ The majority of variceal hemorrhage is related to gastroesophageal varices, with approximately 50% mortality for the initial hemorrhage, but high rates of control, up to 90%, depending on the therapeutic intervention.^10^, ^11^ In spite of the prevalence of caput medusae, massive cutaneous hemorrhage from this area seems extremely rare with only two fatal cases^12^, ^13^ and six other non‐fatal cases^14^, ^15^, ^16^, ^17^, ^18^, ^19^ reported in the literature to our knowledge.

Treatment of this condition includes standard resuscitation protocols as an initial approach in order to obtain hemodynamic stabilization with local measures to control the bleeding during the resuscitation, such as direct pressure, suture ligation, or cautery.^20^, ^21^, ^22^ Other measures in the management of caput medusae hemorrhage are the correction of coagulopathy and the reduction of portal hypertension. Coagulopathy in liver disease results from thrombocytopenia and impaired humoral coagulation. Infusion of fresh frozen plasma (or plasma exchange, in the case of volume overload) is sometimes used but lack proven efficacy with regard to coagulation. Prothrombin complex concentrate is seldom used in patients with liver cirrhosis due to an elevated risk of thrombosis and lack of evidence‐based favorable outcome. Platelet transfusion should be performed to maintain a level above 50 × 10^9^/L.^23^ Lowering portal venous hypertension reduces portosystemic variceal pressure with a resulting reduction in the risk of hemorrhage. Traditional medical therapy with terlipressin, alternatively somatostatin, or analogues (octreotide) is effective on esophageal variceal bleeding and should be empirically applied to umbilical variceal hemorrhage.^24^ Considering that transjugular intrahepatic portosystemic shunt (TIPS) is the most effective interventional treatment for lowering portal hypertension, it is strongly recommended for inpatient care.^25^, ^26^ Patients with caput medusae bleeding have an increased risk of recurrent bleeding within the first few weeks. This patient underwent only suture ligation, and he had a fatal recurrent bleeding after one month. We believe that surgical suture should be considered only as bridging to definitive therapy with TIPS. Other methods of definitive intervention include radiological embolization of the feeding vein of the varix,^16^ or transection procedures.^10^, ^24^ Intravariceal sclerotherapy is not considered as first hand option for umbilical variceal bleeding, because of the poor effect on bleeding control and risk of complications due to high umbilical vein size.

Despite the fact that acute hemorrhage from ectopic cutaneous varices is a very rare medical condition, physicians should address this acute medical situation with similar measures as in the treatment of the more common forms of gastroesophageal variceal bleeding. Initial resuscitation should be performed in order to stabilize hemodynamically unstable patients with aggressive volume supply and parallel hemodynamic monitoring. Local measures with direct pressure, suture ligation, or cautery, and correction of coagulopathy should be applied early in resuscitation. When the patient is hemodynamically stable, definitive therapy should be initiated. TIPS is considered being the cornerstone of interventional treatment. Intensive observation is required, as these patients are at high risk for both complications and recurrent bleeding.

## 4 CONCLUSIONS

Life‐threatening acute hemorrhage from a ruptured caput medusae vein is a rare complication of liver cirrhosis. It is crucial to stop the bleeding promptly. TIPS is considered the most important part of management and potentially lifesaving.

## CONFLICT OF INTEREST

All authors declared no conflict of interest.

## AUTHOR CONTRIBUTION

NM: involved in idea, design, data collection, major writing, and project management. AHY: involved in patient management, and critical revision. PM: involved in writing of the article, and project management. All authors read and approved the final manuscript.

## ETHICAL APPROVAL

All authors approved the final version of the case report for submission to the *Clinical Case Reports*.
